# Metabolomics analysis of aqueous humor from patients with high-myopia complicated nuclear cataract

**DOI:** 10.3389/fmed.2025.1454840

**Published:** 2025-05-21

**Authors:** Qiuyi Huo, Yitong Xu, Yaqi Wang, Shenrong Zhang, Zhexuan Liu, Jin Li

**Affiliations:** ^1^Department of Ophthalmology, Eye Institute, Eye & ENT Hospital, Fudan University, Shanghai, China; ^2^Department of Cataract, Eye Hospital, School of Ophthalmology and Optometry, Wenzhou Medical University, Wenzhou, China

**Keywords:** high-myopia complicated nuclear cataract, aqueous humor, HPLC-MS, metabolites, enrichment analysis

## Abstract

**Background:**

We investigated the metabolic profiles of aqueous humor (AH) among patients with high-myopia complicated nuclear cataract (HMnC), age-related nuclear cataract (NC), cortical cataract (CC), and high myopia (HM); we sought to identify possible metabolic mediators for these conditions.

**Methods:**

The metabolic data of AH from 29 patients (nine with HMnC, nine with CC, seven with NC, and four with high myopia) were analyzed by liquid chromatography-tandem mass spectrometry. Principal component analysis, sample correlation analysis, and orthogonal partial least squares discriminant analysis modeling were conducted. Univariate and multivariate analyses were performed to identify differential metabolites with potential biological significance.

**Results:**

For HMnC patients, the level of glutathione was decreased, whereas arginine, tyrosine, and tryptophan were more abundant in AH. Dihomomethionine and 8-methylthiooctanaldoxime located in the methionine metabolic pathways were downregulated in NC samples compared with HMnC samples. Additionally, the levels of D-alanyl-D-alanine, 1-methylpyrrolinium, L-phenylalanine, ecgonine methyl ester, ecgonine, tropinone, and azacyclohexane, NNK-N-oxide, 3-succinoylpyridine, and N-nitrosodimethylamine were all upregulated in HM samples compared with HMnC samples.

**Conclusion:**

This work identified valuable metabolic biomarkers and pathways that may improve understanding HMnC pathogenesis. Here, we found that a decrease in glutathione might promote the occurrence of HMnC. Arginine, tyrosine, and tryptophan were more abundant in AH from HMnC patients and tended to prevent HMnC progression. These findings have translational value in terms of developing new therapeutic measures for HMnC-related complications.

## 1 Introduction

High myopia (HM) is defined as an axial length (AL) >26 mm or the spherical equivalent of <−6.0 diopters (D). By 2050, 938 million people will have HM, comprising approximately 9.8% of the world’s population (1). HM is a predisposing factor for cataracts (2); high-myopia complicated nuclear cataract (HMnC) exhibits rapid deterioration along with poor preoperative biological detection, surgical complexity, and increased postoperative complications (3). Thus, there is a need to understand the pathogenesis of HMnC for effective prevention and treatment.

Metabolomics is a new field that involves collections of small metabolic molecules (4) to help predict disease risk, identify biomarkers, and assess disease progression (5). Active metabolites rely on chemical modifications or interactions with macromolecules to affect the genome, epigenome, transcriptome, and/or proteome (6, 7). Metabolite abnormalities in aqueous humor (AH) can be used to characterize various ocular diseases (8). In the eye, the lens capsule was bathed by aqueous humor, so AH affected the lens (9). In a gas chromatography/time-of-flight mass spectrometry analysis, Chen et al. (10) found that glycine could serve as a potential biomarker for the early diagnosis of primary congenital glaucoma. Wei et al. (11) observed abnormalities in amino acid, fatty acid, and carbohydrate metabolism in AH in patients with central retinal vein occlusion. Lactate, succinate, 2-hydroxybutyrate, asparagine, dimethylamine, histidine, threonine, and glutamine were identified as metabolites with potential play roles in the development and progression of diabetic retinopathy (12).

Although metabolomics has revealed the metabolic characteristics of common ocular diseases, few studies have concerned the characteristics of AH metabolism in HMnC. To address this knowledge gap, we sought to identify novel metabolite markers for HMnC using AH samples.

## 2 Methods

### 2.1 Clinical sample collection

Twenty-nine patients were included in this study. NC, CC, and HMnC underwent normal cataract surgery and HM without cataract underwent posterior scleral reinforcement surgery. The patients in the HM group do not have cataracts HM. The inclusion criteria for all patients were age ≥18 years, no intraoperative or postoperative complications and AL ≥26 mm in patients with HMnC and patients with HM. There was no requirement for the axial length of patients with CC and patients with NC. Only patients with HMnC were regrouped, and HMnC patients with 26 ≤ AL < 28 mm were assigned to group A, and the remains were assigned to group B (13–15). Patients who met either of the following criteria were excluded from our analysis: (1) presence of strabismus, keratopathy, glaucoma, uveitis, choroidal neovascularization, and/or macular degeneration; (2) presence of hypertension and/or diabetes.

### 2.2 Metabolic profiling and data processing

First, all samples were analyzed by high-performance liquid chromatography-tandem mass spectrometry (HPLC-MS). A Vanquish ultra-high performance liquid chromatograph and a Waters ACQUITY UPLC BEH Amide liquid chromatography columns were used for chromatographic separation of the target compounds. Compound Discover V3.1 software was used to extract data. Compound identification involved online searches of mzCloud and the comprehensive database ChemSpider. The criteria used for the identification of differential metabolites were *t*-test *p*-value <0.05 [or −log10 (*p*-value) >1.3] and VIP >1. Principal component analysis was used for the reduction of data dimensionality through orthogonal transformation. Correlation coefficients between samples were calculated based on the relative expression levels of all metabolites identified by positive and negative ion channels. Finally, orthogonal partial least squares discriminant analysis (OPLS-DA) modeling was conducted to identify differential metabolites with potential biological significance. Metabolic pathway annotation and enrichment analysis of differential metabolites were performed using the Kyoto Encyclopedia of Genes and Genomes Pathway data.^1^ Differential metabolites were annotated using the Human Metabolome Database (HMDB).

### 2.3 Statistical analysis

Statistical analyses were performed with SPSS Statistics 26 software. Differential metabolites were compared among groups using the Student’s *t*-test and analysis of variance. Multivariate statistical analysis was used to compare variable importance in projection (VIP) values for OPLS-DA. The *p*-values in this study will be uniformly expressed as −log10 (*p*-value). −log10 (*p*-value) >1.3 (equal to *p*-value <0.05) was considered statistically significant.

## 3 Results

### 3.1 Clinical data and patient characteristics

The 29 AH samples shown in Table 1 were divided into four groups: high myopia nuclear cataract (HMnC), age-related cortical cataract (CC), age-related nuclear cataract (NC), and high myopia (HM). The sample size, age, and gender of each subgroup were shown in Table 1. The study protocol was approved by the Ethics Committee of the Eye Hospital of Wenzhou Medical University (ID: 2022-009-K-07-01) and conducted following the principles of the Declaration of Helsinki.

**Table 1:** Sample information.

Group	Samples	Ages	Sex (male/female)
High myopia nuclear cataract	HMnC1-9	67.22 ± 15.19	3/6
Age-related cortical cataract	CC1-9	67.67 ± 10.15	3/6
Age-related nuclear cataract	NC1-7	74.14 ± 9.08	3/4
High myopia	HM1-4	53.50 ± 16.34	1/3
HMnC A (26 ≤ axis length < 28 mm)	HMnC2, HMnC4, HMnC5, HMnC8	80.00 ± 9.31	1/3
HMnC B (axis length >28 mm)	HMnC1, HMnC3, HMnC6, HMnC7, HMnC9	57.00 ± 10.12	2/3

### 3.2 Nontargeted metabolomics in AH samples

Table 2 shows the metabolites that had exact matches in multiple databases (mzCloud Search, ChemSpider Search, and MassList Search). In positive ion mode, the number of metabolites was 5,000; 43 had exact matches. The total number of metabolites in negative ion mode was 5,083; 44 had exact matches.

**Table 2:** Compound discover (CD) results for metabolites with exact matches.

Negative CD results	Positive CD results
L-(+)-Lactic acid	DL-Carnitine
citric acid	Acetyl-L-carnitine
DL-Lactic acid	DL-Stachydrine
Pyruvic acid	Creatine
Itaconic acid	Valine
Citric acid	5′-S-Methyl-5′-this adenosine
Levulinic acid	2-Amino-1,3,4-octadecanetriol
Theophylline	Indole-3-acetic acid
DL-Lactic acid	N,N-Diethylethanolamine
Uridine	Propionylcarnitine
Pyruvic acid	DL-Arginine
δ-Gluconic acid δ-lactone	2-Amino-1,3,4-octadecanetriol
2-Oxoglutaric acid	Phenacetin
Citric acid	L-Phenylalanine
2-Oxoglutaric acid	D-(-)-Glutamine
Pyruvic acid	D-Carnitine
Ascorbic acid	Phenacetin
Linoleic acid	Edaravone
Ascorbic acid	Coniine
L-Phenylalanine	4-Guanidinobutyric acid
Linoleic acid	5-Hydroxytryptophan
Salicylic acid	3-(2-Hydroxyethyl) indole
Meso-erythritol	5-Hydroxytryptophan
D-(-)-Glutamine	Coniine
Ascorbic acid	DL-Carnitine
Malonic acid	Acetylarginine
N-Acetylaspartic acid	3-Indoleacetonitrile
D-(+)-Arabitol	Glycylproline
L-Serine	N,N-Diethylethanolamine
Creatine	2-Amino-1,3,4-octadecanetriol
Hippuric acid	N6-Me-adenosine
Threonine	Hexanoylcarnitine
D-(-)-Glutamine	Decanoylcarnitine
Υ-Aminobutyric acid (GABA)	Nicotine
L-Serine	Kojic acid
N-Phenylacetylglutamine	D-(-)-Glutamine
4-Nitrophenol	N-Phenylacetylglutamine
D-(+)-Tryptophan	N4-Acetylcytidine
N-Formylmethionine	Cytidine
L-(-)-Malic acid	Glycyl-L-leucine
Vanillin	N6-Me-adenosine
N-acetylvaline	proline
L-Tyrosine	Paracetamol
N-Acetyl-DL-glutamic acid	

**Figure 1:**
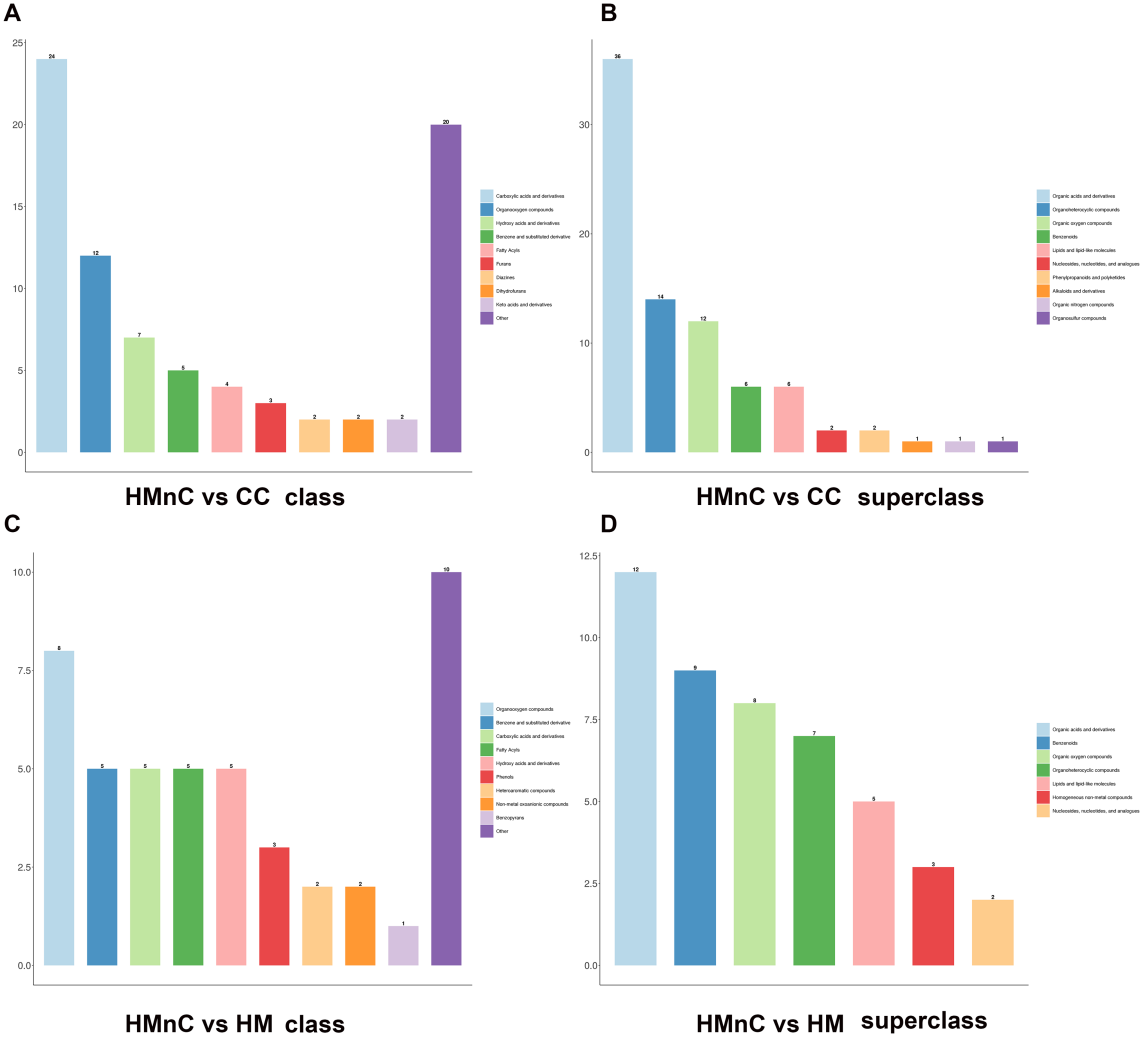
HMDB annotation results based on class and superclass modes were divided into 10 categories. Panels **(A,B)** show HMnC-vs-CC comparisons; panels **(C,D)** show HMnC-vs-HM comparisons.

The HMDB annotation results for metabolites are shown in Figure 1. Figures 1A,B show the HMDB annotation results for HMnC-vs.-CC differential metabolites. Differential metabolites mainly included organic acids and their derivatives, carboxylic acids and their derivatives, organic heterocyclic compounds, and organic oxygen compounds. Figures 1C,D show the HMDB annotation results for HMnC-vs.-HM differential metabolites. Differential metabolites mainly included four categories: organic oxygen compounds, carboxylic acids, and their derivatives, benzenes, and organic acids and their derivatives.

### 3.3 Principal component analysis and OPLS-DA modeling

**Figure 2:**
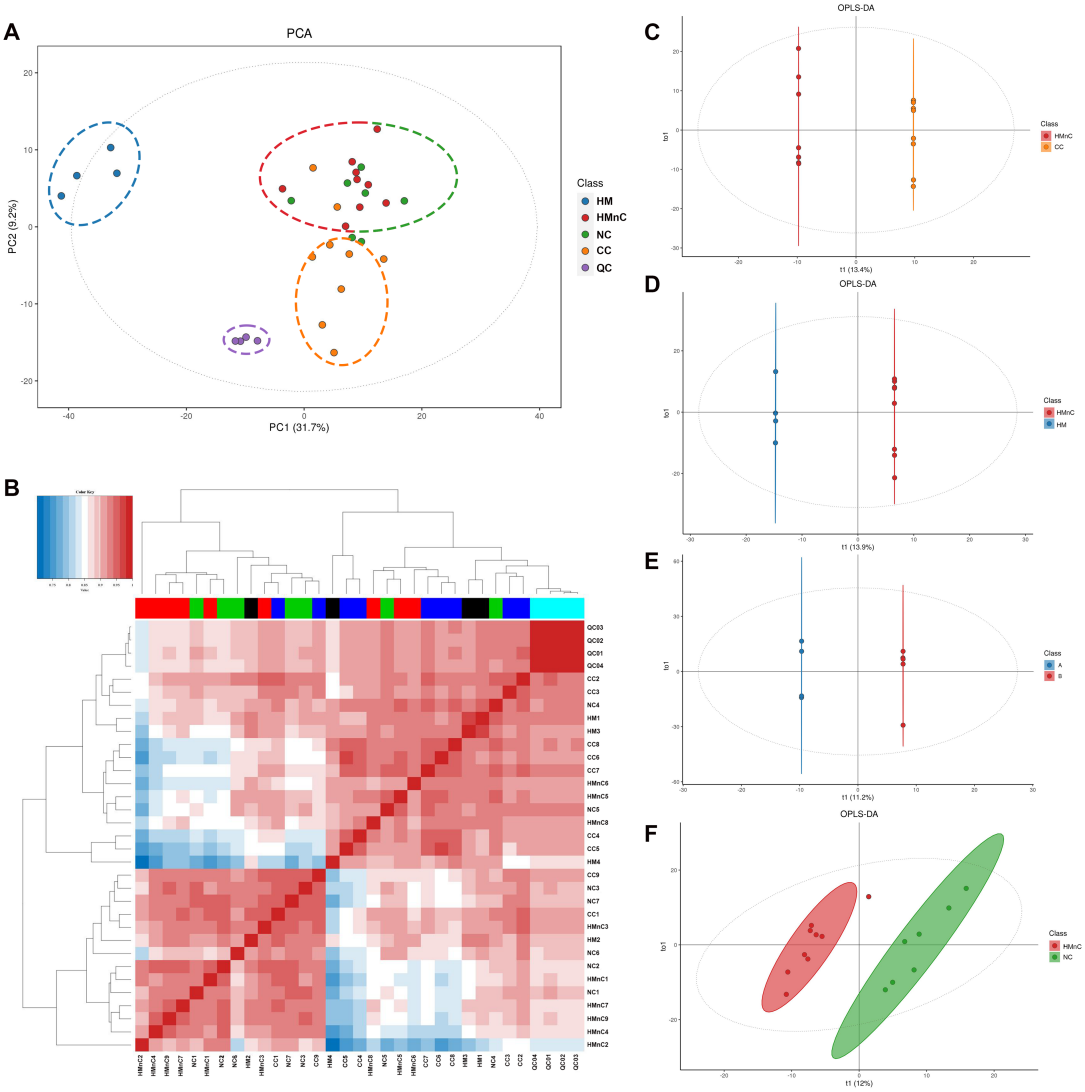
Panel **(A)** shows a principal component analysis scatter plot. Panel **(B)** shows a diagram of the correlation analysis between samples; the gradient from white to blue indicates a negative correlation, whereas the gradient from white to red indicates a positive correlation. Panels **(C–F)** show OPLS-DA modeling scatter plots that compare different groups. T1 represents the predicted principal component score of the first principal component; To1 represents the first orthogonal principal component score.

The samples were divided into four groups as shown in Figure 2A. Samples from patients with HMnC and patients with NC belonged to one group; samples from patients with CC and patients with HM were in other groups, respectively. The groups were separated by principal component analysis; PC1 showed that the HMnC group had a 31.7% difference from the HM group. PC2 showed that the CC group was separated from the other cataract groups (HMnC and NC); this component explained 9.2% of the difference. Figure 2B shows that there were apparent differences in metabolites among the groups. Finally, the HMnC and NC samples were clustered into one group, whereas the CC and HM samples were clustered into another group.

The modeling results obtained using OPLS-DA are shown in Supplementary Table S1. The HMnC group was used as the control group to conduct paired modeling with the HM, NC, and CC groups. Paired modeling was also performed between the HMnC A and HMnC B groups. Except for the comparison between HMnC and NC, the *Q*^2^ value for all group comparisons was >0.6, indicating that the constructed models were reliable. The OPLS-DA score plots (Figures 2C–F) indicated that clear separation occurred among the comparison groups.

### 3.4 Differential metabolite analysis

**Figure 3:**
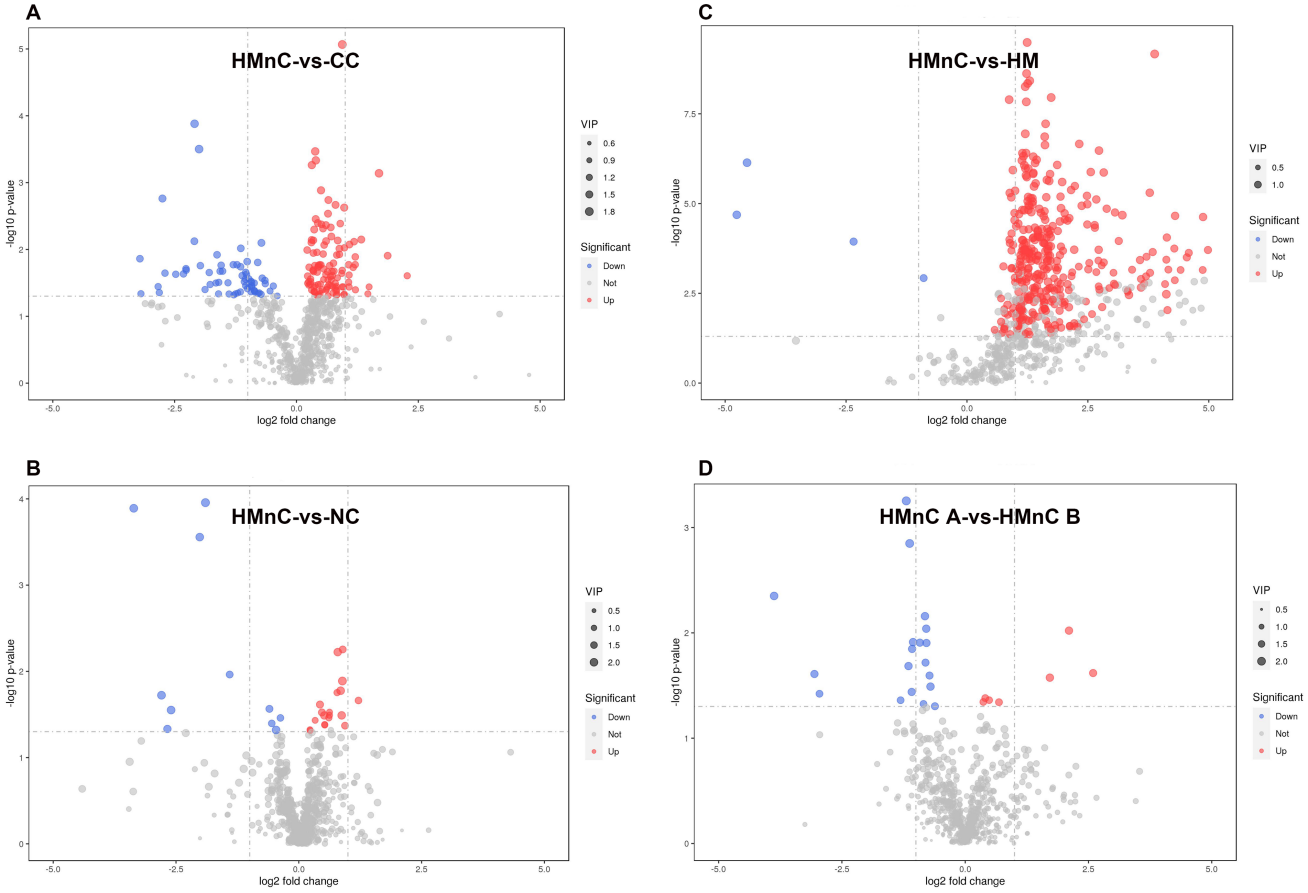
**(A)** HMnC-vs-CC, **(B)** HMnC-vs-NC, **(C)** HMnC-vs-HM, **(D)** A-vs-B. Each point in the volcano plot represents a metabolite; the horizontal axis represents the fold change for each substance in the group (base 2 logarithm), whereas the vertical axis represents the *p*-value from Student’s *t*-test (base 10 negative logarithm). The sizes of the scatter points represent VIP values in the OPLS-DA model; larger scatter points indicate a larger VIP value and greater compound importance. Scattered point colors represent the final screening results, with significantly upregulated metabolites in red, significantly downregulated metabolites in blue, and out-of-threshold metabolites in gray. The selection criteria were *t*-test *p*-value < 0.05 and VIP > 1.

The numbers of metabolic differences are shown in Figure 3. The top 10 upregulated and downregulated differential metabolites are shown in Supplementary Table S2. Compared with CC samples, HMnC samples had 178 differential metabolites (114↑ and 64↓). The upregulated metabolites were guaiacol sulfate, glycerophosphoglycerol, and 2-oxovalericacid; the downregulated metabolites were 5-(2′-carboxyethyl)-4_6-dihydroxypicolinate, d-pipecolicacid, 2-3-4-5-tetrahydrodipicolinate (Supplementary Table S2-1). Compared with HM samples, HMnC samples had 376 differential metabolites (371 ↑ and 4 ↓). Tropicamide and N, N-diethylethanolamine were upregulated; lidocaine N-oxide and N′-hydroxy-4-pentylbenzenecarboximidamide were downregulated (Supplementary Table S2-2). Compared with NC, HMnC samples had 30 differential metabolites (19↑ and 11↓). Sebacic acid and 2-oxovalericacid were upregulated; etilevodopa, 4-phenyl butyric acid, and alpha-glutamyl-4-hydroxyproline were downregulated (Supplementary Table S2-3). Compared with HMnC B group samples, HMnC A group samples had 26 differential metabolites (7↑ and 19↓). p-cresolsulfatepotassium, (2S)-2-piperazinecarboxylic acid, and N-phenylacetylglutamine were upregulated; chorismate and 3_4-dihydroxymandelate were downregulated (Supplementary Table S2-4).

### 3.5 Metabolic pathway annotation and enrichment analysis of differential metabolites

**Figure 4:**
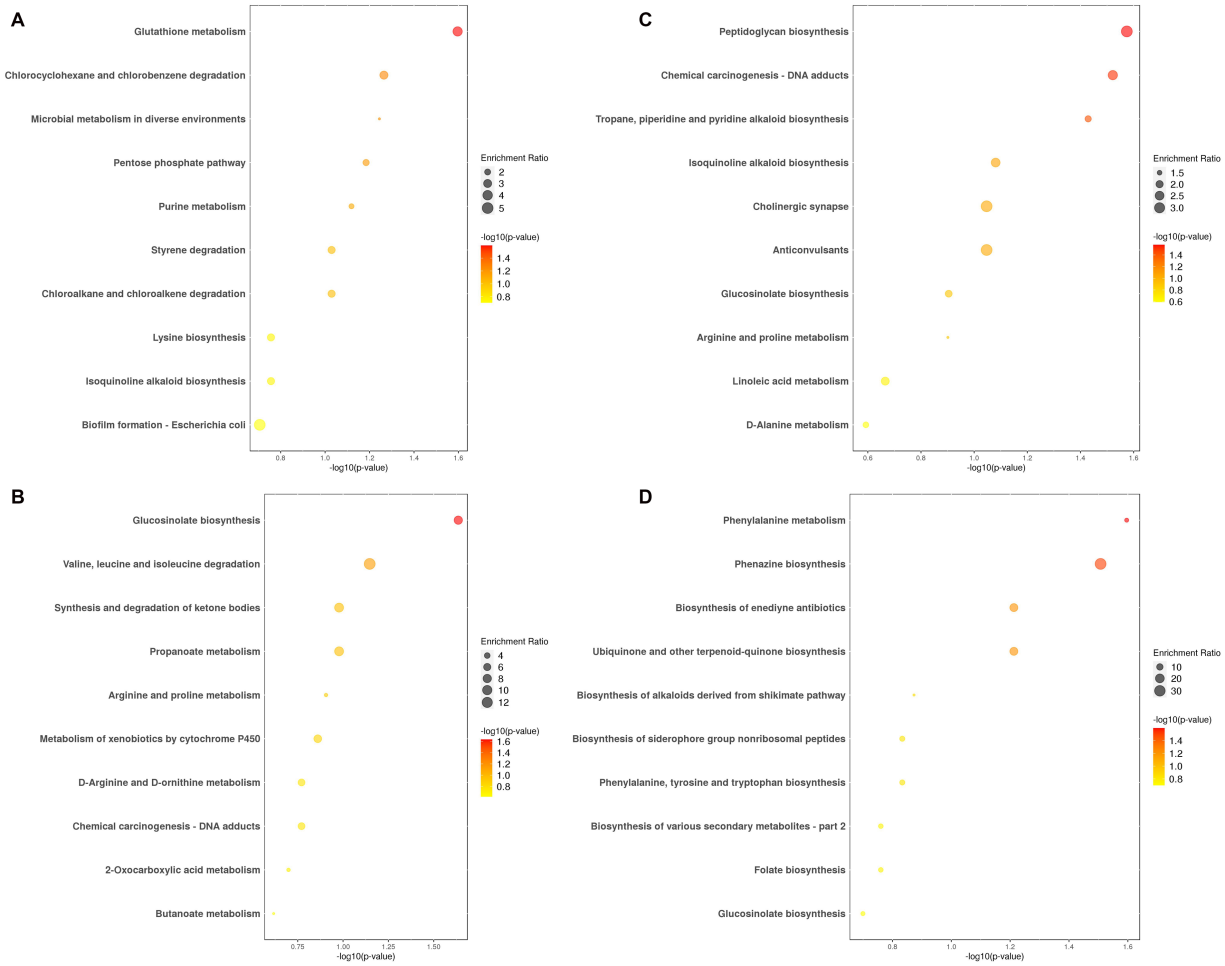
The top 10 annotated and enriched pathways of differential metabolites. **(A)** HMnC-vs-CC, **(B)** HMnC-vs-NC, **(C)** HMnC-vs-HM, **(D)** HMnC A-vs-HMnC B. Each point in the plot represents a pathway; the horizontal axis represents -log10 (*p*-value), whereas the vertical axis represents the pathway name. The sizes of the points represent enrichment ratio; larger points indicate a higher number of enriched genes. The point colors represent the significance of enrichment. The darker the color of the dot, the larger the value of -log10 (*p*-value). The selection criteria were t-test -log10 (*p*-value)> 1.3.

Figure 4A and Supplementary Table S3-1 show the top 10 annotated and enriched pathways of differential metabolites in HMnC samples vs. CC samples. These pathways include glutathione metabolism; chlorocyclohexane and chlorobenzene degradation; microbial metabolism in diverse environments; pentose phosphate pathway; purine metabolism; styrene degradation; chloroalkane and chloroalkene degradation; lysine biosynthesis; isoquinoline alkaloid biosynthesis; biofilm formation—*Escherichia coli*; the pathway with a statistically significant difference was hsa00480 glutathione metabolism (Supplementary Figure S1). Compared to the HMnC, glycine was a significantly upregulated metabolite, whereas ascorbic acid was downregulated in the CC samples.

Figure 4B and Supplementary Table S3-2 show the top 10 annotated and enriched pathways for the differential metabolites in HMnC samples vs. NC samples. These pathways include glucosinolate biosynthesis; valine, leucine, and isoleucine degradation; propanoate metabolism; synthesis and degradation of ketone bodies; arginine and proline metabolism; metabolism of xenobiotics by cytochrome P450; chemical carcinogenesis—DNA adducts; D-arginine and D-ornithine metabolism; 2-oxocarboxylic acid metabolism and butanoate metabolism; the pathway with a statistically significant difference was map00966 glucosinolate biosynthesis (Supplementary Figure S2). Differential metabolites were located in the methionine metabolic pathways. Compared with HMnC samples, dihomomethionine and 8-Methylthiooctanaldoxime were downregulated in NC samples.

Figure 4C and Supplementary Table S3-3 show the top 10 annotated and enriched pathways for the differential metabolites in HMnC samples vs. HM samples. These pathways included peptidoglycan biosynthesis; chemical carcinogenesis_DNA adducts; tropane, piperidine, and pyridine alkaloid biosynthesis; isoquinoline alkaloid biosynthesis; cholinergic synapse; anticonvulsants; glucosinolate biosynthesis; arginine and proline metabolism; linoleic acid metabolism and D-alanine metabolism. Pathways with statistically significant differences included map00550 peptidoglycan biosynthesis, hsa05204 chemical carcinogenesis_DNA adducts, and map 00960 tropane, piperidine, and pyridine alkaloid biosynthesis (Supplementary Figure S3). The differential metabolite in map00550 is located in the amino sugar and nucleotide sugar metabolism. Compared with HMnC samples, the levels of D-alanyl-D-alanine is upregulated in HM samples. The differential metabolites in map00960 are located in the arginine and proline metabolic pathways and the phenylalamine, tyrosine, and tryptophan metabolic pathways. Compared with HMnC samples, the levels of 1-methylpyrrolinium, L-phenylalanine, ecgonine methyl ester, ecgonine, tropinone, and azacyclohexane were all upregulated in HM samples; the differential metabolite in hsa05204 is centered in N-nitroso compounds. Compared with HMnC samples, the levels of NNK-N-oxide, 3-succinoylpyridine; N-nitrosodimethylamine were upregulated in HM samples.

Figure 4D and Supplementary Table S3-4 show the top 10 annotated and enriched pathways for the differential metabolites in HMnC A group samples vs. HMnC B group samples. These pathways included phenylalanine metabolism; phenazine biosynthesis; biosynthesis of enediyne antibiotics; ubiquinone and other terpenoid-quinone biosynthesis; biosynthesis of alkaloids derived from the shikimate pathway; phenylalanine, tyrosine, and tryptophan biosynthesis; biosynthesis of siderophore group nonribosomal peptides; folate biosynthesis; biosynthesis of various secondary metabolites_part 2 and glucosinolate biosynthesis. Pathways with statistically significant differences included hsa00360 phenylalanine metabolism and map00405 phenazine biosynthesis (Supplementary Figure S4). We found differential metabolites in the phenylalanine biosynthesis and metabolism pathways. Compared with HMnC A group samples, N-acetyl-L-phenylalanine and phenylacetylglutamine were upregulated in HMnC B group samples, whereas chorisate was downregulated.

## 4 Discussion

Metabolomics is a powerful method for studying pathophysiological processes. Using HPLC-MS for metabolomics analysis allows for high sensitivity and resolution in detecting a wide range of metabolites in complex biological samples. The combination of mzCloud and ChemSpider databases enhances metabolite identification accuracy and efficiency, offering extensive spectral and chemical information that surpasses traditional databases. Many researches also used HPLC-MS, mzCloud, and ChemSpider databases for analysis (16–18). This integrated approach supports more reliable biomarker discovery and metabolic profiling in research. In our future studies, we also consider using other databases or methods for cross-validation to improve the reliability of the results. The KEGG database provides a systematically curated repository of canonical metabolic pathways, enzyme functions, and molecular networks, offering robust annotation capabilities. Previous researchers, Kanehisa et al. (19), also used KEGG databases for functional genomics and pathway-centric analyses. Cross-validation with alternative pathway databases (e.g., MetaCyc) represents a robust methodological strategy to enhance the reliability and biological plausibility of pathway annotations in untargeted metabolomics studies.

Cataract pathogenesis involves changes in lens protein expression (20) and amino acid structure (21); increased oxidative stress damage and changes in enzyme activity (22); epigenetic methylation of antioxidant genes (23), genetic mutations (24), and the intraocular inflammatory microenvironment activated after surgery (25). In this study, we found that glycine upregulation caused an increase in glutathione; the glutathione level was significantly lower in HMnC samples than in CC samples, suggesting that a decrease in the glutathione level might promote the occurrence of NC in patients with HM. Differentially phosphorylated sites have been associated with glutathione metabolism in patients with HM (26). Moreover, levels of glutathione reportedly differ according to cataract type, and glutathione rapidly decreases in patients with NC (27). Our findings were consistent with the results of previous studies concerning the glutathione level in the HMnC group.

Furthermore, we found that the levels of 1-methylpyrroline, L-phenylalanine, ecgonine methyl ester, ecgonine, tropinone, and piperidine were all upregulated in HM samples, compared with HMnC samples; these changes promoted the metabolism of the arginine, tyrosine, amino acid and tryptophan. Therefore, the levels of these amino acids were higher in the HMnC group. In a previous study, samples from patients with HM exhibited metabolite abundance and metabolic changes (8). The most abundant metabolites in AH from patients with HM were aminocaprylic acid, arginine, citrulline, and dihydrosphingosine (28). Distinct lens proteins are cross-linked by tryptophan and tyrosyl radicals in advanced NC lenses (29). A disturbance in tryptophan metabolism has been associated with cataract formation (30). Increases in tryptophan, tyrosine, carnitine, and glycerophosphate in the lenses of OXYS rats were attributed to a compensatory response to oxidative stress (31). Our research results were consistent with the previous findings about the higher levels of amino acids in the HMnC group.

The levels of cysteine-related metabolites exhibited the following pattern (from high to low): HM, HMnC B, HMnC A, and NC. Notably, these levels were positively correlated with axial length. In groups with longer axial length, the phenylalanine metabolic pathway was upregulated, whereas the synthetic pathway was downregulated. Homocysteine and methionine levels are higher in myopic rodent eyes than in normal rodent eyes (32). Decreased levels of dopamine and its metabolites, along with slower biosynthesis, were observed in a chick model of myopia, suggesting that retinal dopamine is involved in the regulation of eye axis growth (33). Feldkaemper hypothesized that dopamine constitutes a “stop” signal for axial growth (34). Form deprivation myopia and lens-induced myopia could be inhibited by topical application of levodopa (35, 36). The results of our study were consistent with the results of previous studies about the relationship between axial length and phenylalanine metabolic pathway.

This work had some limitations. First, we only recruited 29 eligible patients; this small sample size might have led to bias in the subgroup analysis. Second, although this study suggested that glutathione reduction could induce NC in HM, the metabolomic comparison of CC and NC lacked robust evidence. Third, model stability might have affected the experimental analysis. Considering the implications of our results, we are recruiting additional patients and collecting more detailed clinical data to validate our metabolomic findings.

## 5 Conclusion

Patients with HMnC had an AH metabolomic profile distinct from the profiles in patients with CC and patients with NC. Compared with patients with HM, arginine, tyrosine, and tryptophan levels were increased in AH samples from patients with HMnC. A decrease in glutathione might induce NC in patients with HM. The levels of methionine-related metabolites were positively correlated with eye axis growth, whereas the level of phenylalanine was negatively correlated with eye axis growth. Routine assessments of aqueous metabolomic profiles in patients undergoing posterior scleral reinforcement surgery may help to assess the risk of subsequent NC.

## Data Availability

The original contributions presented in the study are included in the article/Supplementary material, further inquiries can be directed to the corresponding authors.

## Footnotes

^1^ www.kegg.jp/kegg/pathway.html
