# Supplementary material for: Metabolomics analysis of aqueous humor from patients with high-myopia complicated nuclear cataract
Source: Front Med (Lausanne). 2025 May 21;12:1454840. doi: 10.3389/fmed.2025.1454840 (PMC12133728; doi:10.3389/fmed.2025.1454840)
Supplement: Supplementary file 1 [file Data_Sheet_1.PDF]

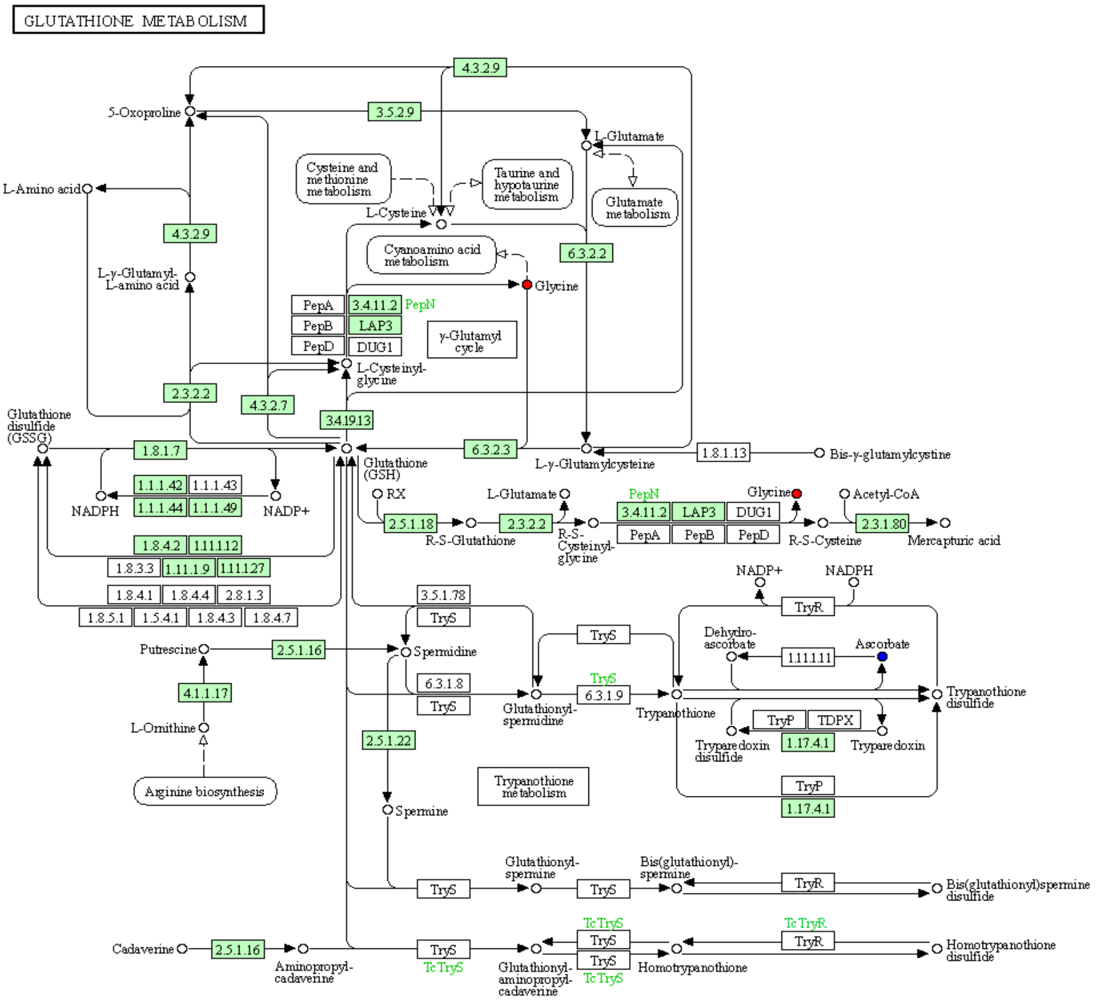

Figure S1 glutathione metabolic pathway diagram (HMnC-vs-CC)

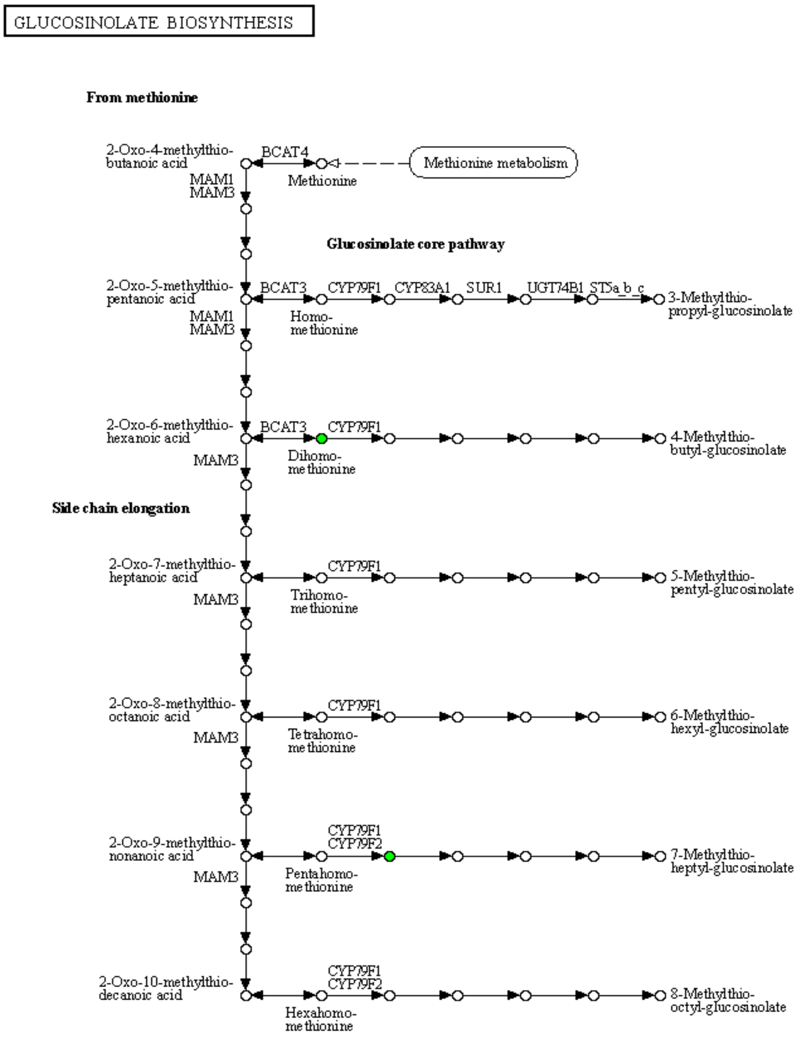

FigureS2. glucosinolate synthesis pathway diagram. (HMnC-vs-NC)

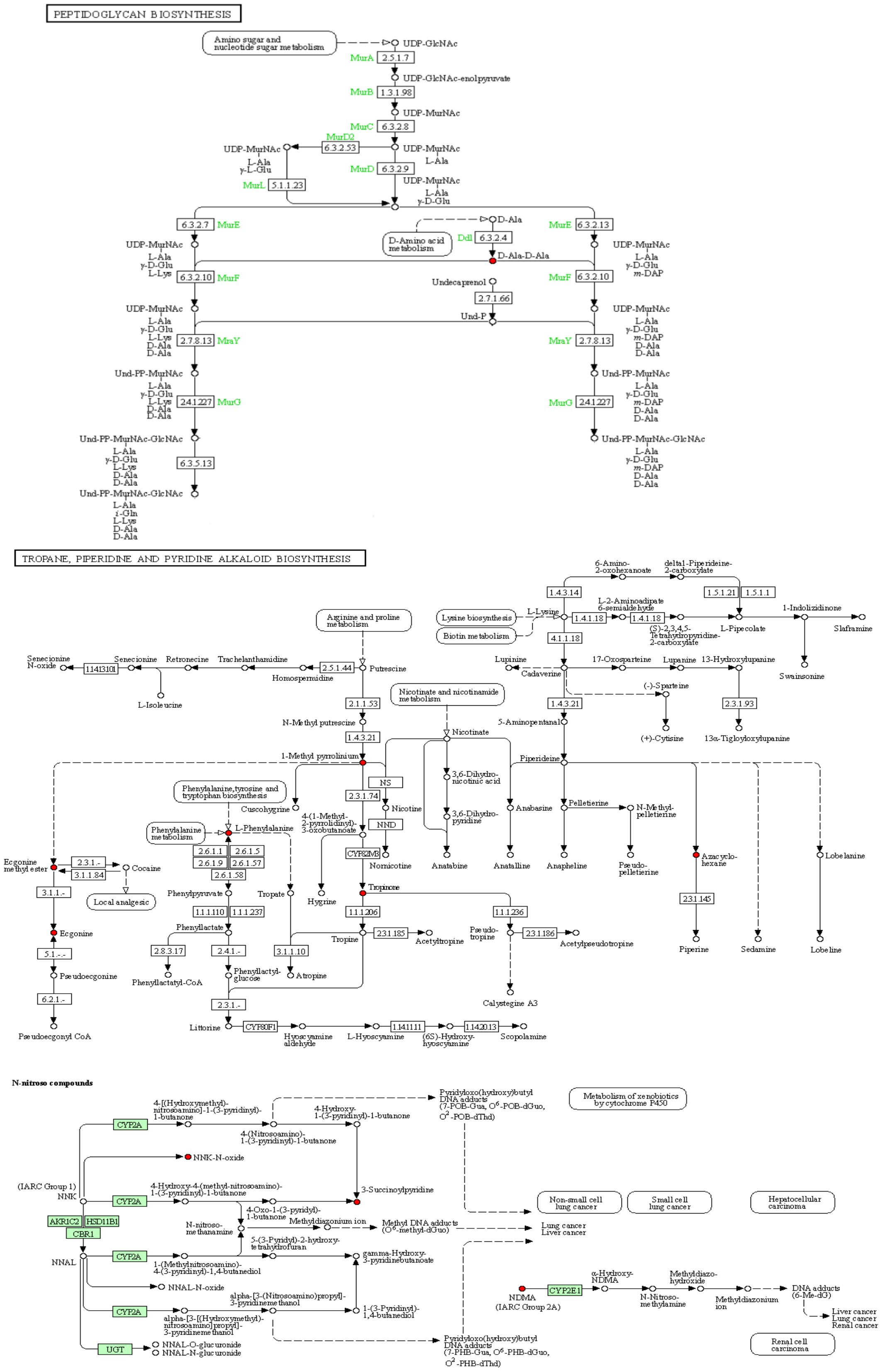

Figure S3 peptidoglycan biosynthesis pathway diagram, tropane, piperidine, and pyridine alkaloid biosynthesis pathway diagram, and chemical carcinogenesis _DNA

adducts pathway diagram. (HMnC-vs-HM）

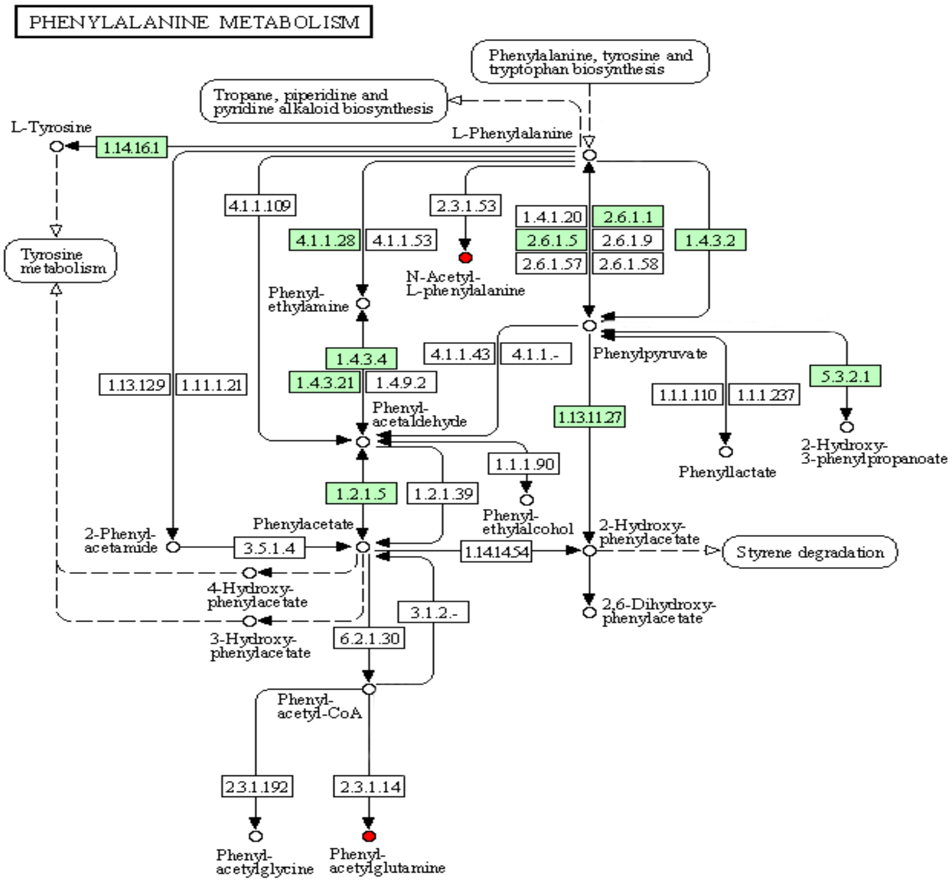

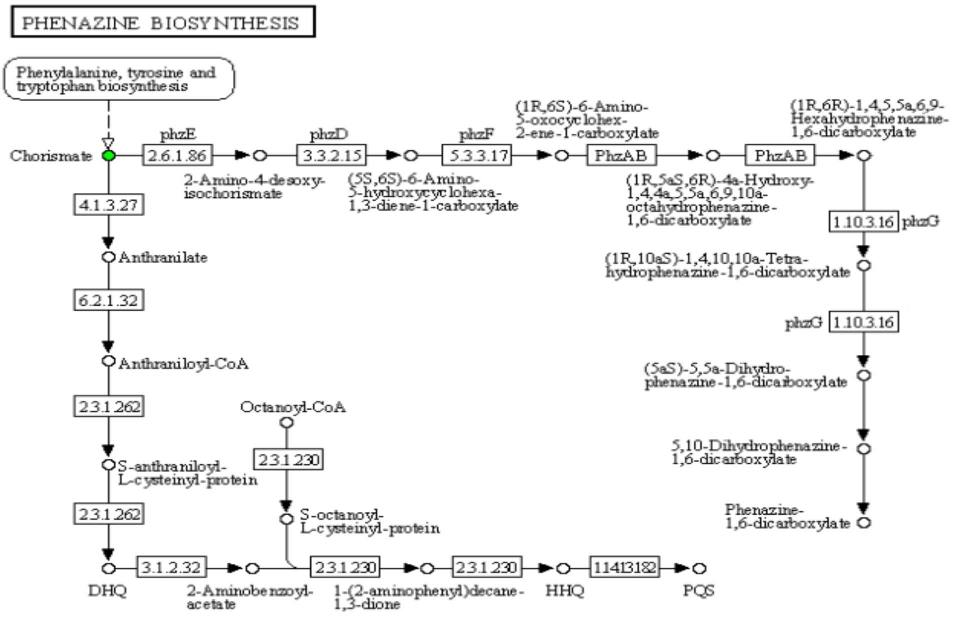

Figure S4 phenylalanine metabolism pathway diagram, phenazine biosynthesis pathway diagram（HMnC A-vs-HMnC B）

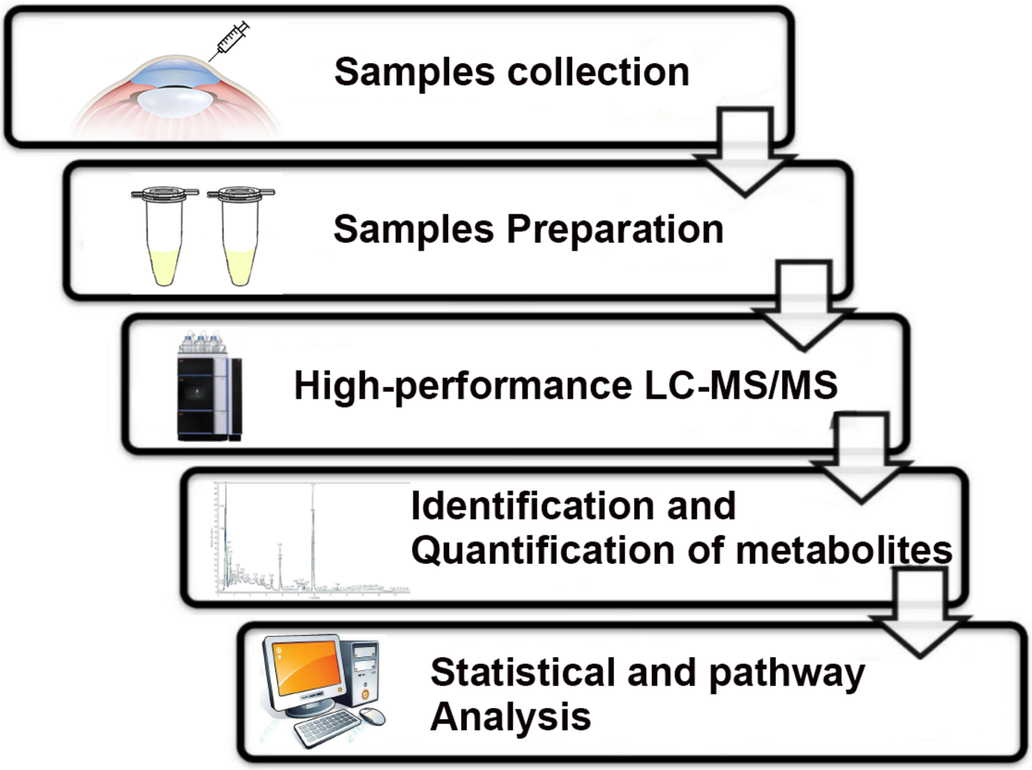

Figure S5 Summary cartoon

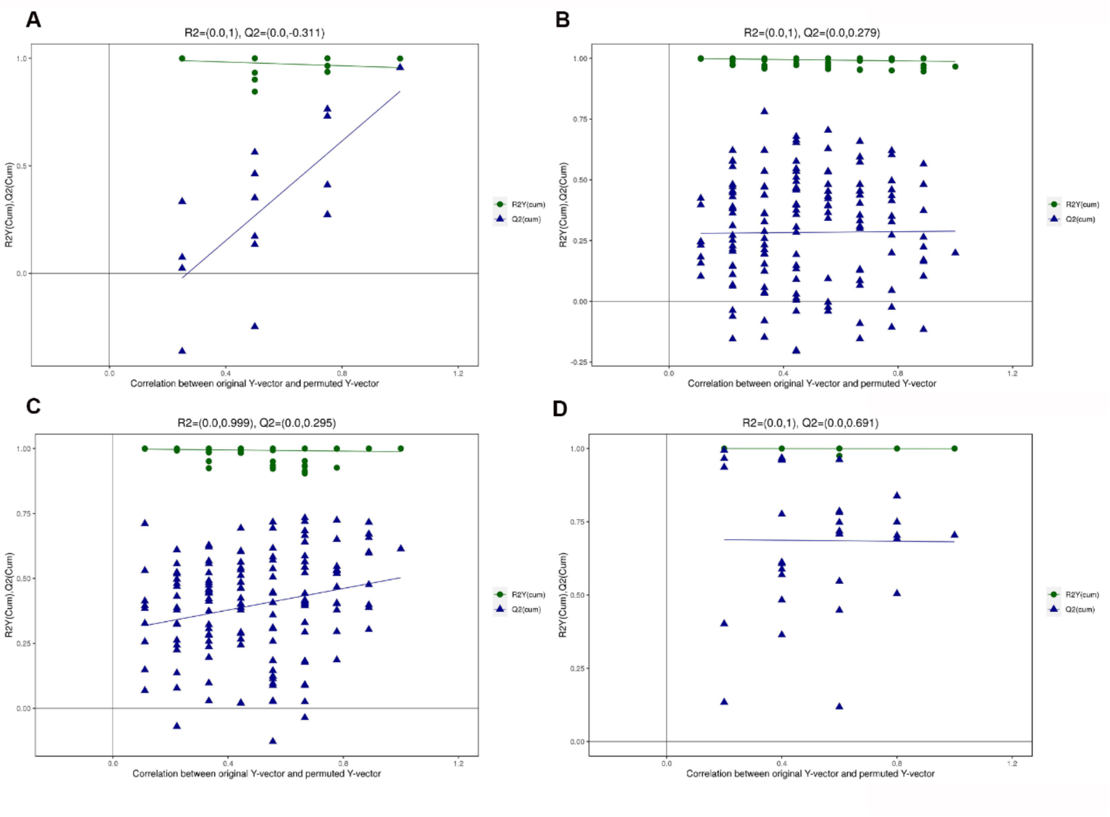

Figure S6 Permutation test results of OPLS-DA model.

A: HMnC vs HM; B: HMnC vs NC; C: HMnC vs CC; D: HMnC Avs HMnC B
