# Supplementary material for: Metabolomics analysis of aqueous humor from patients with high-myopia complicated nuclear cataract
Source: Front Med (Lausanne). 2025 May 21;12:1454840. doi: 10.3389/fmed.2025.1454840 (PMC12133728; doi:10.3389/fmed.2025.1454840)
Supplement: Supplementary file 2 [file Data_Sheet_2.PDF]

Table S1 OPLS-DA modeling parameters and results

**Title**	**R^2X(cum)**	**R^2Y(cum)**	**Q^2(cum)**
**HMnC vs HM**	0.609	0.999	0.957
**HMnC vs NC**	0.487	0.983	0.0286
**HMnC vs CC**	0.694	1	0.614
**HMnC Avs HMnC B**	0.854	1	0.704

Table S2 Compound Discover (CD) results for metabolites with exact matches

Neg CD results

Name	Formular	Molecular	RT [min]	RT Tolerance	Area (Max)
pyruvic acid	$C_3 H_4O_3$	88.01607	1.357	0.174	455441334.8
δ-gluconic acid δ-lactone	$C_6H_{10}O_6$	178.04783	1.248	0.156	425346797.2
2-oxoglutaric acid	$C_5H_6O_5$	146.02156	0.772	0.044	418208866.3
citric acid	$C_6H_8O_7$	192.02716	2.718	0.2	388039099
2-oxoglutaric acid	$C_5H_6O_5$	146.02157	0.955	0.044	375080297.1
pyruvic acid	$C_3H_4O_3$	88.01603	1.447	0.174	297987121.1
ascorbic acid	$C_6H_8O_6$	176.03217	1.844	0.2	104054320.5
linoleic acid	$C_{18}H_{32}O_2$	280.24049	0.578	0.032	98119923.47
ascorbic acid	$C_6 H_8O_6$	176.03218	1.319	0.103	78653888.12
l-phenylalanine	$C_9H_{11}NO_2$	165.07904	4.524	0.2	73464433.78
linoleic acid	$C_{18}H_{32}O_2$	280.2404	0.696	0.032	70864617.88
salicylic acid	$C_7H_6O_3$	138.03169	0.665	0.2	43690432.52
meso-erythritol	$C_4H_{10}O_4$	122.05805	2.682	0.2	39620009.68
D-(-)-glutamine	$C_5H_{10}N_2O_3$	146.06927	6.409	0.049	22279693.32
ascorbic acid	$C_6H_8O_6$	176.03216	1.452	0.103	19629253.15
malonic acid	$C_3H_4O_4$	104.01098	0.543	0.2	17872302.54
N-acetylaspartic acid	$C_6H_9NO_5$	175.04822	6.81	0.2	17072162.84
D-(+)-arabitol	$C_5H_{12}O_5$	152.06862	4.01	0.046	9769980.484
L-serine	$C_3H_7NO_3$	105.04269	1.897	0.2	8210558.842
creatine	$C_4H_9N_3O_2$	131.06957	5.986	0.2	7782952.413
hippuric acid	$C_9H_9NO_3$	179.05852	3.381	0.2	6881707.994
threonine	$C_4H_9NO_3$	119.0584	6.068	0.2	6584791.782
D-(-)-glutamine	$C_5H_{10}N_2O_3$	146.06924	6.65	0.2	6073861.702
Y-aminobutyric acid (GABA)	$C_4H_9NO_2$	103.06344	5.57	0.2	5641170.467
L-serine	$C_3H_7NO_3$	105.04266	0.654	0.097	3935407.126
N-phenylacetylglutamine	$C_{13}H_{16}N_2O_4$	264.11135	3.981	0.2	3786907.686
4-nitrophenol	$C_6H_5NO_3$	139.02711	0.557	0.2	3484943.596
D-(+)-tryptophan	$C_{11}H_{12}N_2O_2$	204.09	4.576	0.2	3335065.273
N-formylmethionine	$C_6H_{11}NO_3S$	177.04619	3.42	0.2	3315059.785
L-(-)-malic acid	$C_4H_6O_5$	134.02169	7.436	0.2	2165018.138
vanillin	$C_8H_8O_3$	152.04744	0.577	0.2	2132843.536
N-acetylvaline	$C_7H_{13}NO_3$	159.08966	3.675	0.2	1976237.445
L-tyrosine	$C_9H_{11}NO_3$	181.07403	5.242	0.2	1455385.888
N-acetyl-DL-glutamic acid	$C_7H_{11}NO_5$	189.06392	6.701	0.2	1424473.806

Pos CD results

Name	Formular	Molecular	RT [min]	RT Tolerance	Area (Max)
DL-arginine	$C_6H_{14}N_4O_2$	174.11111	8.692	0.2	154959324
2-amino-1,3,4-octadecanetriol	$C_{18}H_{39}NO_3$	317.29176	0.657	0.2	154793016
phenacetin	$C_{10}H_{13}NO_2$	179.09408	1.025	0.2	147840604
L-phenylalanine	$C_9H_{11}NO_2$	165.07847	4.527	0.2	143392823
D-(-)-glutamine	$C_5H_{10}N_2O_3$	146.0687	6.408	0.2	124966031
D-carnitine	$C_7H_{15}NO_3$	161.10469	5.525	0.2	122282391
phenacetin	$C_{10}H_{13}NO_2$	179.09406	1.322	0.2	78658869.6
edaravone	$C_{10}H_{10}N_2O$	174.07869	0.923	0.2	69252751
coniine	$C_8H_{17}N$	127.13565	3.023	0.067	65277473.1
4-guanidinobutyric acid	$C_5H_{11}N_3O_2$	145.08465	6.19	0.2	63689273.3
5-hydroxytryptophan	$C_{11}H_{12}N_2O_3$	220.08407	0.799	0.07	61697941.8
3-(2-hydroxyethyl) indole	$C_{10}H_{11}NO$	161.08342	1.029	0.2	60194611.5
5-hydroxytryptophan	$C_{11}H_{12}N_2O_3$	220.08414	0.686	0.07	59744956.1
coniine	$C_8H_{17}N$	127.13565	3.348	0.084	32802356.6
DL-carnitine	$C_7H_{15}NO_3$	161.10468	5.226	0.2	32115420.2
acetylarginine	$C_8H_{16}N_4O_3$	216.12158	6.337	0.2	29804701
3-indoleacetonitrile	$C_{10}H_8N_2$	156.06817	0.561	0.2	25075322.6
glycylproline	$C_7H_{12}N_2O_3$	172.08427	5.49	0.2	23850549.5
N, N-diethylethanolamine	$C_6H_{15}NO$	117.11491	3.77	0.057	22229861
2-amino-1,3,4-octadecanetriol	$C_{18} H_{39} NO_3$	317.29189	2.355	0.2	15510842.4
N6-Me-Adenosine	$C_{11}H_{15}N_5O_4$	281.1116	4.912	0.2	15283027.9
hexanoylcarnitine	$C_{13}H_{25}NO_4$	259.1776	4.012	0.2	14882687.6
decanoylcarnitine	$C_{17}H_{33}NO_4$	315.24023	3.519	0.2	11648781.1
nicotine	$C_{10}H_{14}N_2$	162.11522	1.047	0.2	11146197
kojic acid	$C_6H_6O_4$	142.02619	1.255	0.2	8170427.35
D-(-)-glutamine	$C_5H_{10}N_2O_3$	146.06882	5.56	0.2	7425866.89
N-phenylacetylglutamine	$C_{13}H_{16}N_2O_4$	264.11028	3.987	0.2	7177894.33
N4-acetylcytidine	$C_{11}H_{15}N_3O_6$	285.09528	2.75	0.2	5942913.47
cytidine	$C_9H_{13}N_3O_5$	243.08478	4.149	0.2	5763040.62
glycyl-L-leucine	$C_8H_{16}N_2O_3$	188.11548	4.33	0.031	5386911.9
N6-Me-adenosine	$C_{11}H_{15}N_5O_4$	281.11155	1.985	0.2	3425835.47
proline	$C_5H_9NO_2$	115.06293	1.557	0.2	2507370.88
4-guanidinobutyric acid	$C_5H_{11}N_3O_2$	145.08469	5.57	0.2	1906268.9
paracetamol	$C_8H_9NO_2$	151.06305	4.246	0.2	1712965.57

Table S3 Statistical table of differential metabolite results

S3-1. HMnC vs. CC

Name	VIP	FC	P
guaiacol sulfate	1.236645	4.830924	0.024749
glycerophosphoglycerol	1.412694	3.658032	0.012397
2-oxovalericacid	1.71364	3.234355	0.000725
4-ethyl-2,6-dihydroxy phenyl hydrogen sulfate	1.173331	2.809494	0.036296
medorinone	1.181813	2.760598	0.046161
dokdolipid A	1.491853	2.513656	0.007102
sapacitabine	1.245454	2.31342	0.040153
10-hydroxydec-2-enoic acid	1.355687	2.307448	0.012915
caprylic acid	1.335214	2.304796	0.016904
3-indoxyl sulfate	1.38152	2.268982	0.007644

Name	VIP	FC	P
5-(2'-carboxyethyl)-4_6-dihydroxypicolinate	1.383128	0.107903	0.013753
d-pipecolic acid	1.24811	0.109493	0.045936
2_3_4_5-tetrahydrodipicolinate	1.266189	0.139773	0.035955
2_5-dihydroxypyridine	1.254822	0.142147	0.04416
etilevodopa	1.523477	0.148642	0.00173
4-nitrocatechol	1.331013	0.154104	0.022603
nitrosoguvacoline	1.312784	0.179621	0.023503
phenobarbital	1.332939	0.200461	0.023208
N2-acetyl-L-aminoadipate	1.354678	0.207759	0.019391
trimethadione	1.345474	0.208208	0.02039

S3-2 HMnC vs HM

Name	VIP	FC	P
tropicamide	1.2585446	14403.98	1.33E-05
N, N-diethylethanolamine	1.2501541	539.7335	2.14E-05
phenylephrine	1.201627	205.2468	3.12E-05
(+)-10-deoxymethynolide	1.146955	202.1436	0.000209
arg-his	1.0538168	169.2094	0.000891
2_5_6-trihydroxy-5_6-dihydroquinoline	1.1696965	156.4635	0.000184
3-(2-hydroxyethyl)indole	1.1276688	148.2076	0.000231
carteolol	1.1218228	129.9888	0.000241
metamfepramone	1.0428693	116.4347	0.000945
5-allyl-5-sec-butyl-1,3-dimethyl-2,4,6(1H,3H,5H)-pyrimidinetrione	1.1265383	94.73223	0.000254

Name	VIP	FC	P
lidocaine N-oxide	1.2878154	0.022379	1.45E-05
N'-hydroxy-4-pentylbenzenecarboximidamide	1.2970822	0.042769	7.27E-07
rasagiline	1.1249029	0.19689	0.000115
(±)-laudanosine	1.0663126	0.537522	0.001191

S3-3. HMnC vs. NC

Name	VIP	FC	P
sebacic acid	1.514125	2.320928	0.02177
2-oxovalericacid	1.587119	1.919408	0.042638
1-(3_4-dimethoxy phenyl) ethane-1_2-diol	1.612685	1.857937	0.005571
N-gondoylethanolamine	2.108071	1.849798	0.01294
4-(stearoylamino)butanoic acid	1.972189	1.83074	0.032381
N-(11Z,14Z)-eicosadienoylethanolamine	2.012834	1.804319	0.016769
N-octadecanoyl-L-homoserine lactone	1.949283	1.731544	0.005979
3-[(3-hydroxytridecanoyl)oxy]-4-(trimethylammonium)butanoate	1.35365	1.716183	0.017629
(R)-3-hydroxybutyrate	1.204189	1.542215	0.029712
12-hydroxydodecanoicacid	1.29674	1.540128	0.032278

Name	VIP	FC	P
etilevodopa	2.074294	0.097363	0.000129
4-phenyl butyric acid	2.08785	0.144061	0.018918
alpha-glutamyl-4-hydroxyproline	1.857678	0.156559	0.04653
(2S)-3-Phenyl-2-({[(3S,4S,5R)-2,3,4-trihydroxy-5-(hydroxymethyl)tetrahydro-2-furanyl]methyl}amino)propanoic acid (non-preferred name)	2.041358	0.164958	0.028105
isophthalic acid	1.993117	0.247086	0.000278
5-hydroxyferulate	2.196228	0.267864	0.000111

[1-(3,4-dihydroxy-5-methoxyphenyl)-7-(3,4-dihydroxy phenyl)heptan-3-yl] acetate	1.601112	0.376996	0.010884
hydrogen bromide	1.663282	0.660708	0.027218
3,6,9,12-tetraoxatridec-1-yl methacrylate	1.507349	0.682702	0.040127
methylmalonic acid	2.026768	0.72559	0.047824

S3-4. HMnC A vs. HMnC B

Name	VIP	FC	P
p-cresolsulfatepotassium	1.750996	6.036635	0.024075
(2S)-2-piperazinecarboxylic acid	1.818557	4.300507	0.00952
N-phenylacetylglutamine	1.831422	3.286769	0.02662
N-acetyl-L-phenylalanine	1.616825	1.60741	0.045517
salsolinol	1.562657	1.40008	0.043523
5-hydroxy-2-oxo-4-ureido-2_5-dihydro-1h-imidazole-5-carboxylate	1.617114	1.325658	0.041605
2-methyl-3-hydroxy-5-formylpyridine-4-carboxylate	1.586939	1.290147	0.045268

Name	VIP	FC	P
chorismate	1.918002	0.068146	0.004463
3_4-dihydroxymandelate	1.785465	0.120116	0.02457
N2-acetyl-L-aminoadipate	1.598565	0.403367	0.043616
(2E)-5-(methylsulfanyl)-2-[(methylsulfanyl)methyl]-2-pentenal	2.090678	0.436784	0.000558
cis-aconiticacid	1.828603	0.450583	0.020664
(2R)-3-sulfolactate	2.024285	0.458126	0.001416
[fahydroxy(20:3)]11_12-dihydroxy-5Z_8Z_14Z-eicosatrienoicacid	1.785504	0.471345	0.036386
acetylene dicarboxylate	1.784071	0.474133	0.014206
(4S)-4_6-dihydroxy-2_5-dioxohexanoate	1.860732	0.48071	0.01224

Table S4 Metabolic pathway annotations of differential metabolites

Table S4-1 HMnC-vs-CC

Pathway ID	PathwayName	p-value
**hsa00480**	glutathione metabolism	0.025339
**map00361**	chlorocyclohexane and chlorobenzene degradation	0.054375
**map01120**	microbial metabolism in diverse environments	0.056973
**hsa00030**	pentose phosphate pathway	0.06541
**hsa00230**	purine metabolism	0.07609
**map00625**	chloroalkane and chloroalkene degradation	0.093496
**map00643**	styrene degradation	0.093496
**map00950**	isoquinoline alkaloid biosynthesis	0.175228
**map00300**	lysine biosynthesis	0.175228
**map02026**	biofilm formation - Escherichia coli	0.196891

Table S4-2 HMnC-vs-NC

Pathway ID	PathwayName	p-value
**map00966**	glucosinolate biosynthesis	0.023152
**hsa00280**	valine, leucine and isoleucine degradation	0.071314
**hsa00640**	propanoate metabolism	0.105173
**hsa00072**	synthesis and degradation of ketone bodies	0.105173
**hsa00330**	arginine and proline metabolism	0.124161
**hsa00980**	metabolism of xenobiotics by cytochrome P450	0.137882
**hsa05204**	chemical carcinogenesis - DNA adducts	0.169478
**map00472**	D-arginine and D-ornithine metabolism	0.169478
**hsa01210**	2-oxocarboxylic acid metabolism	0.199937
**hsa00650**	butanoate metabolism	0.241467

Table S4-3 HMnC-vs-HM

Pathway ID	PathwayName	p-value
**map00550**	peptidoglycan biosynthesis	0.0266490310207927
**hsa05204**	chemical carcinogenesis - DNA adducts	0.0300915876233448
**map00960**	tropane, piperidine, and pyridine alkaloid biosynthesis	0.037248618001287
**map00950**	isoquinoline alkaloid biosynthesis	0.0830086005425735
**map07033**	anticonvulsants	0.0897651571226701
**hsa04725**	cholinergic synapse	0.0897651571226701
**map00966**	glucosinolate biosynthesis	0.124671884854377
**hsa00330**	arginine and proline metabolism	0.125537226707486
**hsa00591**	linoleic acid metabolism	0.215997409326425
**map00473**	D-alanine metabolism	0.255440284372554

Table S4-4 HMnC A-vs-HMnC B

Pathway ID	PathwayName	p-value
**hsa00360**	phenylalanine metabolism	0.025344
**map00405**	phenazine biosynthesis	0.031088
**hsa00130**	ubiquinone and another terpenoid-quinone biosynthesis	0.061288
**map01059**	biosynthesis of enediyne antibiotics	0.061288
**map01063**	biosynthesis of alkaloids derived from the shikimate pathway	0.133887
**hsa00400**	phenylalanine, tyrosine, and tryptophan biosynthesis	0.146787
**map01053**	biosynthesis of siderophore group nonribosomal peptides	0.146787
**hsa00790**	folate biosynthesis	0.17366
**map00998**	biosynthesis of various secondary metabolites - part 2	0.17366
**map00966**	glucosinolate biosynthesis	0.199755
